# Potential of Grape Extract in Comparison with Therapeutic Dosage of Antibiotics in Weaning Piglets: Effects on Performance, Digestibility and Microbial Metabolites of the Ileum and Colon

**DOI:** 10.3390/ani11102771

**Published:** 2021-09-23

**Authors:** Emina Rajković, Christiane Schwarz, David Tischler, Karl Schedle, Nicole Reisinger, Caroline Emsenhuber, Vladimira Ocelova, Nataliya Roth, Dörte Frieten, Georg Dusel, Martin Gierus

**Affiliations:** 1 FFoQSI GmbH—Austrian Competence Centre for Feed and Food Quality, Safety and Innovation, 3430 Tulln, Austria; emina.rajkovic@ffoqsi.at (E.R.); david.tischler@students.boku.ac.at (D.T.); 2 Department of Agrobiotechnology, Institute of Animal Nutrition, Livestock Products, and Nutrition Physiology (TTE), IFA-Tulln, University of Natural Resources and Life Sciences, Vienna (BOKU), 1190 Vienna, Austria; karl.schedle@boku.ac.at (K.S.); martin.gierus@boku.ac.at (M.G.); 3 BIOMIN Research Center, 3430 Tulln, Austria; Nicole.Reisinger@dsm.com (N.R.); caroline.emsenhuber@dsm.com (C.E.); 4 BIOMIN Holding GmbH, 3131 Getzersdorf, Austria; Vladimira.Ocelova@dsm.com (V.O.); nataliya.roth@dsm.com (N.R.); 5 Department of Animal Nutrition, University of Applied Sciences, 55411 Bingen am Rhein, Germany; doerte.frieten@thuenen.de (D.F.); g.dusel@th-bingen.de (G.D.)

**Keywords:** grape extract, tannins, polyphenols, weaning piglets, antibiotic treatment, phytogenic feed additives, apparent total tract digestibility, performance, microbial metabolites, *Vitis vinifera*

## Abstract

**Simple Summary:**

Diarrhea as a symptom of different enteric infections leads to poor animal health and performance at weaning, followed by economic losses. Phytogenic feed additives, e.g., grape extracts, have shown antimicrobial and anti-inflammatory properties and these might have beneficial effects on growth trends of weaning piglets and, thereby, potentially reduce the need for antibiotic treatments following weaning. An 8-week feeding trial investigated the potential effects of grape extract (GE) in a model with a negative control (NC) and positive control (PC; antibiotic treatment). Despite no changes in animal performance, dietary GE improved the digestibility of selected nutrients at the same, or even at higher level, as PC. Additionally, there was no clear effect of dietary intervention on the microbial metabolites from the ileum and colon at the end of the trial. These results indicated beneficial effects of GE compared to antibiotic treatment, as often applied at weaning.

**Abstract:**

Enteric diseases in piglets, such as post-weaning diarrhea (PWD), often require antibiotic treatment of the entire litter. Grape polyphenols may help overcome PWD and thereby reduce the need for antibiotics. The potential of a grape extract (GE; continuous in-feed supplementation) on performance of weaning piglets, compared with both negative (NC; corn-based diet) and positive control (PC; NC + in-feed antibiotic (amoxicillin) in a therapeutic dosage for day 1–day 5 post weaning) was assessed. Apparent total tract digestibility (ATTD) and microbial metabolites were also evaluated on two sampling points (day 27/28 and day 55/56). We assigned 180 weaning piglets (6.9 ± 0.1 kg body weight (BW)) to 6 male and 6 female pens per treatment with 5 piglets each. Animals from PC showed higher BW on day 13 compared with NC and GE, and a tendency for higher BW on day 56 (*p* = 0.080) compared to NC. Furthermore, PC increased the average daily feed intake in the starter phase (day 1–day 13), and the average daily gain in the early grower phase (day 14–day 24). Overall, GE improved the ATTD at the same level as PC (ash, acid-hydrolyzed ether extract), or at a higher level than PC (dry matter, organic matter, gross energy, crude protein, P). There were no effects on microbial metabolites apart from minor trends for lactic acid and ammonia. Dietary inclusion of GE may have beneficial effects compared to therapeutic antibiotics, as frequently used at weaning.

## 1. Introduction

Enteric diseases in the post-weaning phase present a serious challenge for pig producers, and lead to impaired animal health and performance, with increased piglet mortality [1] and consequently increased economic losses [2]. Weaning piglets, with their still immature gastrointestinal tract, are highly susceptible to gastrointestinal disorders, including disorders of the gut barrier, immune function and enteric nervous system, which frequently result in post-weaning diarrhea (PWD) [3,4]. PWD is often associated with different pathogenic enterotoxigenic *E. coli* (ETEC) serotypes and *Salmonella* [1,5]. In practice, different antibiotics are frequently applied prophylactically or metaphylactically, to avoid outbreaks of enteric infections in animal stocks [6]. However, antibiotics not only affect intestinal pathogens but may also impair the commensal intestinal microbiota, leading to a dysbiosis in the gut [7,8]. In addition, the often uncontrolled and widespread usage of antibiotics has led to a rapid rise of antibiotic resistance worldwide, which compromises human and animal health [9,10]. Significant reduction of pro- and metaphylactic antibiotic treatments is of high priority for the European Commission. There is an urgent requirement to establish measures for a more prudent usage of antibiotics in veterinary medicine [11], especially for those antibiotics critically important for humans such as amoxicillin [12]. The respective regulations including intensified restrictions for usage of pro- and metaphylactic antibiotic treatments will come into force from January 2022 in the European Union [13]. Amoxicillin (a broad-spectrum antibiotic) became one of the most used antibiotics for treating different systemic infections in pigs, including gastrointestinal infections, by inhibiting the synthesis of bacterial cell walls [1,14]. Antibiotics are used very often prophylactically or metaphylactically at weaning, applying the dosage and duration that is recommended for therapeutic usage, e.g., with infections of gastrointestinal tract. Aside from this usage, antibiotic growth promoters (AGP; subtherapeutic concentrations of antibiotics, fed continuously; banned in many countries since 2006), have been used in livestock diets due to their growth promoting properties (Regulation of European Commission No 1831/2003). Research efforts on antibiotic alternatives over the past years have been based mainly on the experiences with AGP with or without microbial challenge [15,16]. However, a broader approach in scientific research is needed to investigate the impact of available alternatives in comparison with the antibiotic treatments often applied prophylactically or metaphylactically at weaning to relieve PWD.

The need to reduce antibiotics use in food-producing animals [9] has led to increased comprehensive research on feed additives with efficient properties to maintain gut health of piglets during weaning, thereby further supporting animal performance. Phytogenic feed additives (PFA) are plant-derived products, and they have received great attention in recent years as supplements for maintaining or enhancing performance in monogastrics [17,18,19]. These properties are possibly related to the effects on the gastrointestinal microbial community and activity as well as enhanced digestibility [20], through their antimicrobial, antioxidant and anti-inflammatory properties [17,21,22]. However, inconsistent results about the impact of PFA on the intestinal microbiota [23,24] indicate the complexity in selecting PFA. Among PFA, polyphenols are assumed to exert strong antimicrobial [25], anti-inflammatory and antioxidant effects [26,27]. Besides their chemical structure, composition and mode of feeding the polyphenols, the final spectrum of activity of polyphenols will also be determined by digesta content, including specific interaction of polyphenols with the contained microbiota [19]. Polyphenols from grape have been recognized for their inhibitory potential on gram-positive and some gram-negative bacteria such as *E. coli* [28] and the potential to modulate intestinal microflora by promoting commensal beneficial bacteria [29,30]. Several attempts have been made to assess and explain the mode of action of grape polyphenols, mostly with broilers, by diet supplementation with grape by-products from different parts of grape: grape extract (GE), grape pomace, grape skin, grape seed and grape seed extract [31,32,33,34,35]. Results in the literature suggest that the concentration of some polyphenols have a saturation limit, above which the effects of polyphenols might be restricted or even become adverse.

Local beneficial effects of polyphenols and their metabolites in the large intestine may occur by advancing the colonocytes, even if not absorbed [36], since more than 95% of some polyphenols from grape (tannins) enter the colon undigested [37]. It can be assumed that the presence of luminal phenolic compounds and the stimulation of intestinal fermentation will increase the generation of microbial metabolites from endogenous and dietary compounds. However, depending on the type, the contribution of microbial metabolites to host physiology may be both adverse and beneficial [38]. Hence, we hypothesized that GE polyphenols can positively affect the performance of piglets—in the context of an unforeseen diarrhea outbreak post weaning—at least to the same level of effectiveness as an initial prophylactic or metaphylactic antibiotic treatment. Therefore, in a feeding trial we evaluated the effect of GE in a diet of weaning piglets in comparison with both negative (NC, without antibiotic treatment) and positive control (PC, with antibiotic treatment) on performance parameters. Our focus is directed to the antimicrobial effects of polyphenols, assessing possible changes in the concentration of microbial metabolites. In searching for the mode of action of GE, a comparison between the effects of GE and PC on the selected parameters enables us to highlight where GE acts in a similar manner as PC and where the mode of action may differ.

## 2. Materials and Methods

The experimental procedures in this study were conducted in strict accordance with the German Animal Welfare Act and were approved by the relevant Department for Animal Welfare Affairs (Landesuntersuchungsamt Rheinland-Pfalz, Koblenz, Germany; registration no. 23 177-07/G 18-20-021).

### 2.1. Experimental Design, Animals, and Housing

An 8-week feeding trial was conducted using weaning piglets (DanBred × Piétrain) with an average initial body weight (BW) of 6.9 ± 0.1 kg. Two days before the start of the trial 180 piglets were individually identified using ear tags, weighed, and selected for the trial. At weaning (23 ± 1 day of age), piglets were allotted to 36 pens according to BW and sex in a randomized complete block design. Each pen contained 5 piglets to receive one of 3 experimental diets, with 6 male and 6 female pen replicates per treatment. Three pens started the trial with 4 instead of 5 piglets, since 3 selected piglets (NC = 2; GE = 1) were not delivered to the trial facility.

The experimental treatments were as follows: (1) a corn-based control diet [negative control, NC]; (2) NC + amoxicillin for the first 5 days of the trial [positive control, PC]; (3) NC + grape extract (150 g/t of feed; according to supplier’s recommendation) [GE] for the whole trial. Oral antibiotic amoxicillin (Amoxicillin-Trihydrate 100, 1000 mg/g, powder for oral application, Bela-Pharm GmbH & Co. KG, Vechta, Germany) was administered according to dosage recommendations for piglets with gastrointestinal infections (20 mg amoxicillin/kg BW every 12 h, for 5 days). The respective amount of antibiotic was mixed with the feed and applied in a feeder, the dosage was therefore calculated according to the average BW and number of piglets per pen. The grape extract was dried (water-based) extract from dried grapes (*Vitis vinifera*, total polyphenols >40%, procyanidins >30%, water <5%, BIOMIN Holding GmbH, Getzersdorf, Austria).

The piglets were housed in strawless pens with a slatted floor. Each pen was equipped with a manual feed trough and one nipple drinker, providing *ad libitum* access to feed and water throughout the experiment. The room temperature was set at 32 °C in the first week of the trial and was gradually decreased to 24 °C in a linear manner until the end of the trial.

### 2.2. Experimental Diets

The diets were formulated to meet or exceed the nutrient requirements for piglets according to the German Society for Nutrition Physiology [39]. The composition and calculated nutrient concentration of the control diets are shown in Table 1. Animals received corn-based basal diets in ground form in the starter (day 1–day 13) and grower (day 14–day 56) phase. For a better adaptation to the ground diet, the experimental diets mixed with water in a separate feeder were additionally provided (day 1–day 7). The experimental additive in the PC group was added to the diet in a separate feeder (twice a day, on day 1–day 5), after the feeder had been emptied and cleaned.

All treatment groups received 0.5% ground maize premix in the starter and grower phases. In the GE group, a powder of GE was added to the maize premix (see footnote 2, Table 1). In grower diets, titanium dioxide (TiO_2_) was added at a rate of 0.3% as an indigestible marker to allow determination of apparent total tract digestibility (ATTD).

### 2.3. Growth Performance

The individual BW of each piglet and feed intake per pen were measured weekly to determine the average daily feed intake (ADFI), average daily gain (ADG) and feed conversion ratio (FCR).

### 2.4. Euthanasia

At two points during the trial, i.e., T1 (4 weeks post weaning, day 27/28; 11.8 ± 1.7 kg BW) and T2 (8 weeks post weaning, day 55/56; 25.2 ± 0.8 kg BW), one representative piglet of average BW per pen was euthanized. The piglets were first sedated by an intra-venous injection (V. jugularis externa) of 0.5 mL/10 kg BW of azaperone (Stresnil, 40 mg/mL, Elanco GmbH, Cuxhaven, Germany) and 2 mL/10 kg BW of ketamine (Ursotamine, 100 mg/mL, Serumwerk Bernburg AG, Bernburg, Germany). Ten minutes later, after deep sedation was confirmed (total absence of palpebral/corneal and pedal withdrawal reflex response), the piglets were euthanized by intracardiac injection of 6 mL/10 kg BW of T61^®^ (MSD Animal Health GmbH, Unterschleißheim, Germany). Immediately afterwards, piglets were weighed, the abdominal cavity was opened, and the gastrointestinal tract was ligated at the oesophageal region of the stomach and at the rectum. Subsequently, the gastrointestinal tract was removed from the abdomen and prepared for sampling.

### 2.5. Sampling

Representative feed samples were collected at the beginning of the starter and grower phases. Prior to faeces collection, the pens were cleaned. Pooled samples of faeces were collected from each pen twice a day on two consecutive days (day 25/26 and day 53/54) before the respective euthanasia to determine the ATTD.

For ileal and colonic digesta sampling at T1 and T2, the small and large intestine was identified and isolated. The distal end of the ileum was opened 2 cm proximal to the ileocecal valve and total luminal content was collected. The colon was opened at the flexura centralis and ~80 g of luminal content was collected. All samples were homogenized and stored immediately at −20 °C until further analyses.

### 2.6. Analyses

Analyses of all samples were performed at least in duplicate. The samples were thawed and homogenized, and subsamples were taken for further processing.

#### 2.6.1. Chemical Analyses

Faeces samples were freeze-dried, and both the feed and faeces samples were ground through a 0.5-mm sieve before analysis. Diets were analyzed for dry matter (DM), ash, crude fibre (CF), acid hydrolyzed ether extract (HEE), sugar and starch, according to the official methods of VDLUFA [40]. Crude protein content (CP; total nitrogen × 6.25) was detected using the Dumas method [41]. After previous wet ashing in a microwave oven (CEM Mars 6, CEM Corporation, Matthews, NC, USA), calcium (Ca) content was determined by flame atomic absorption spectrophotometry (AAnalyst200, Perkin Elmer Inc., Waltham, MA, USA) and phosphorus (P) content was determined spectro-photometrically (Tecan Austria GmbH, Grödig, Austria) using the vanado-molybdate method to measure colour intensity at 436 nm. Gross energy was measured in a bomb calorimeter (IKA-Kalorimeter C200, IKA Werke GmbH & Co. KG, Staufen, Germany) under oxygen atmosphere. In grower diets, TiO_2_ was analyzed based on the method of Jagger et al. [42] and read spectro-photometrically at 405 nm (Shimadzu UV 1800, Shimadzu Corporation, Kyoto, Japan). Additionally, DM content of digesta and DM, CP, ash, HEE, gross energy, Ca, P and TiO_2_ contents of faeces samples were determined. Due to a limited amount of faeces samples, ashed samples were used for Ca and P analysis, after microwave-assisted pressure digestion as described in the official methods of VDLUFA [40]. All diets were analyzed for total phenols contents and condensed tannins contents. Condensed tannins were determined according to a method of Terril et al. [43] and total phenols with the Folin–Ciocalteu-method [44], both with adjustments [45].

The apparent total tract digestibility of DM, organic matter (OM), ash, CP, HEE, gross energy, Ca and P was calculated using the following equation:

ATTD (%) = {100 − [(N_faeces_/N_diet_) × (Ti_diet_/Ti_faeces_)] × 100} (1)

where N_faeces_ is the nutrient concentration in the faeces (g/kg); N_diet_ is the nutrient concentration in the diet (g/kg); Ti_diet_ is the TiO_2_ concentration in the diet (g/kg); Ti_faeces_ is the TiO_2_ concentration in the faeces (g/kg).

The procedures for analysis of ileal and colonic microbial metabolites were as follows. Intestinal contents of the ileum and colon were analyzed for lactic acid (LA), ammonia, biogenic amines (BA) and short chain fatty acid (SCFA) concentrations.

The SCFA concentration was determined by applying the method of Zhao et al. [46]. The digesta sample (~1 g) was weighed and 1 mL of 5 mM internal standard solution (2-ethylbutyric acid 99%, Sigma Aldrich, Steinheim, Germany) was transferred to reach a final concentration of 1 mM. After addition of 4 mL of ddest water, the tubes were thoroughly mixed and put for 1 h on a shaker (POLYMAX 1040, Heidolph Instruments, Schwabach, Germany). Afterwards, HCl (5 M) was used for a pH adjustment to 2–3. Subsequently, the tubes were centrifuged at 3215× *g* for 20 min (5810R, Eppendorf, Wesseling-Berzdorf, Germany) after 10 min of incubation. The supernatant was immediately stored in 2 mL tubes at −20 °C. For analysis, the samples were thawed and centrifuged at 12,066× *g* for 20 min (minispin, Eppendorf, Wesseling-Berzdorf, Germany). Acetic acid, propionic acid, isobutyric acid, butyric acid, isovaleric acid, valeric acid, isocapronic acid, heptanoic acid and hexanoic acid were separated and quantified using a gas chromatographic system (Agilent Technologies 7890A-G3440A-GC System, Agilent Technologies, Santa Clara, CA, USA) with a C-18 capillary column (Agilent CP-Sil 5CB, length 25 m, diameter 0.32 mm) by a flame ionization detection unit (FID).

For the LA and ammonia analyses, a digesta sample (~1 g) was weighed and mixed with 1 mL of perchloric acid (1 M). After 10 min of incubation, 8 mL of ddest water was added, mixed and shaken for 1 h (POLYMAX 1040, Heidolph Instruments, Schwabach, Germany). The tubes were then centrifuged at 3215× *g* for 10 min (5810R, Eppendorf, Wesseling-Berzdorf, Germany) and the supernatant was immediately stored in 2 mL tubes at −20 °C. Thawed samples were centrifuged at 12,066× *g* for 5 min (minispin, Eppendorf, Wesseling-Berzdorf, Germany) and a supernatant fluid was used for the further analysis of LA and ammonia.

The LA concentration was analyzed according to the method described by Pryce [46] with minor modifications. Briefly, a blend of 100 µL sample or standard solution (Lithium L-lactate, Sigma Aldrich, Steinheim, Germany) and 3.9 mL of precipitating reagent was mixed and centrifuged for 5 min at 3215× *g* (5810R, Eppendorf, Wesseling-Berzdorf, Germany). Further steps were carried out with 0.5 mL of supernatant liquid, 3.0 mL sulfuric acid and 50 µL p-hydroxybiphenyl. The LA concentration was measured spectro-photometrically (Tecan Austria GmbH, Grödig, Austria) at 565 nm.

For ammonia concentration the samples were diluted prior to analysis. A mixture of 0.5 mL of salicylate-nitroprusside colour reagent (blend of equal parts of sodium hydroxide 0.3 M, ddest. H_2_O and salicylate-nitroprusside solution) and 0.25 mL of dichloroisocyanurate solution (0.050 g dichloroisocyanurate dissolved in 50 mL ddest. H_2_O) was prepared and 1.0 mL of sample extract or standard solution (ammonia standard solution ROTI^®^Star, Karlsruhe, Germany) was added immediately (within 30 s) for a proper coloring reaction. After 1.5 h of dark incubation at room temperature, the concentration of ammonia was analyzed spectro-photometrically (Tecan Austria GmbH, Grödig, Austria) at 660 nm.

The BA concentration was analyzed according to the method of Saarinen [47], with modifications. Briefly, 8 mL of perchloric acid (0.4 M) was transferred into a tube containing ~1 g of digesta sample and 1 mL of internal standard solution (Heptylamin, Fluka, Buchs, Switzerland), mixed and remained on a shaker (POLYMAX 1040, Heidolph Instruments, Schwabach, Germany) for 1 h. The tubes were incubated at 4 °C over-night, mixed for 30 s and shaken for 1h. After repeated mixing, the tubes were centrifuged at 3215× *g* for 20 min (5810R, Eppendorf, Wesseling-Berzdorf, Germany). The supernatant was immediately stored in 2 mL tubes at −20 °C. For analysis, the samples were thawed and centrifuged at 12,066× *g* for 20 min (minispin, Eppendorf, Germany). A total of 400 µL of standard or sample solution was added to 80 µL of NaOH (1 M) and 125 µL of saturated sodium bicarbonate solution. After mixing, 500 µL of the derivatization reagent (1% dansyl chloride in acetone, prepared daily) was added, mixed and incubated at 40 °C for 45 min. Then, 25 µL of ammonia was added and the sample was mixed and incubated again at room temperature for 60 min. Finally, 840 µL of acetonitrile was added, mixed, and centrifuged for 10 min at 12,066× *g* (minispin, Eppendorf, Wesseling-Berzdorf, Germany) and 1 mL of the supernatant was transferred to HPLC autosampler vials. The concentrations of ethanolamine, methylamine, pyrrolidine (colon digesta only), putrescin, cadaverine, histamine, spermidine and spermine were quantified using reverse-phase HPLC (Waters 2695 Separations Module, Waters, Milford, MA, USA). An RP-18 column (Phenomex inertClone, 5 μm, 250 × 4, 6 mm) was used to perform the separation of the compounds and the detection by their retention times was performed at a wavelength of 254 nm using a UV detection unit (Waters 2489 UV Detector, Waters, Milford, MA, USA). Ammonia-acetate buffer (pH 5) (Carl Roth GmbH + Co. KG, Karlsruhe, Germany) and acetonitrile (HPLC grade; Carl Roth GmbH + Co. KG, Karlsruhe, Germany) were used as eluents. Data calculation was performed using the Empower 3 software (Waters, Milford, MA, USA).

#### 2.6.2. Statistical Analysis

All parameters were analyzed using the MIXED procedure of SAS 9.4. (SAS Institute, Cary, NC, USA). Pen was the experimental unit for ADFI, FCR and ATTD. For ADG and microbial metabolites, where data were collected from a single animal, the experimental unit was the piglet. The statistical model for performance data included treatment group, week and sex as fixed effects. For the statistical analyses, the interaction between the treatment groups, trial weeks and sex were included in the model. Performance data were analyzed separately for the respective phases (starter day 1–day 13, early grower day 14–day 24, late grower day 25–day 56) and over the whole trial duration (day 1–day 56). Using weekly data (pen for ADFI and FCR, animal for ADG), piglet performance parameters were analyzed as repeated measures using the MIXED procedure. The model used for performance data was:

Y_ijkl_ = µ +α_i_ + β_j_ + δ_k_ + (αβ)_ij_ + (αδ)_ik_ + (βδ)_jk_ + (αβδ)_ijk_ + ε_ijkl_

where: Y_ijkl_ = observation, µ = population mean, α_i_ = effect of the treatment group i (i = NC, PC, GE), β_j_ = effect of the sex j (j = male, female), δ_k_ = effect of the trial week k (k = 1, 2, 3, 4, 5, 6, 7, 8) and (αβ)_ij_ = interaction between treatment group and sex effect, (αδ)_ik_ = interaction between treatment group and week effect, (βδ)_jk_ = interaction sex and week effect, (αβδ)_ijk_ = interaction between treatment group, sex and week effect and ε_ijkl_ = residual error.

The statistical model for chemical data included treatment group, sampling point and sex as fixed effects. For the statistical analyses, the interaction between the treatment groups, sampling point and sex was included in the model. Chemical data were analyzed separately for the respective sampling points (T1 and T2, depicting the interaction of treatment and sampling point) and overall (mean of T1 and T2, depicting the main effect of treatment). The model used for chemical analyses was the same as the model mentioned above with one exception: the effect of the trial week k (δ_k_; k = 1, 2, 3, 4, 5, 6, 7, 8) was replaced by the effect of the sampling point k (δ_k_; k = 1, 2).

The least square means (LS-means) were compared by the Tukey–Kramer Test and differences were considered statistically significant at *p* < 0.05 or as trends for *p*-values between 0.05 and 0.10. The tables present LS-means with their pooled standard errors of means (SEM).

## 3. Results

In the first 3 weeks of the trial, there were noticeable clinical symptoms of diarrhea, weakness and inappetence present in all pens. The diarrhea symptoms started on day 7 of the trial. In sum, 15 weaning piglets died during this trial. Mortality was recorded on a daily basis and affected all treatment groups (NC = 4; PC = 4; GE = 7). In addition, eight animals were excluded from the trial because of a necessary antibiotic treatment (sum of diseased and excluded animals per group: 3 of NC, 3 of PC, 2 of GE). Seven animals (NC = 3; PC = 1; GE = 3) that had received non-steroidal antiphlogistic medication were not excluded from the trial, but these animals were not selected for euthanasia. On day 11, pooled faeces samples of 4 pens with clinical symptoms of diarrhea were taken for analysis by an external lab and found positive, inter alia, on ETEC strain K88. The isolates of *Escherichia coli* were tested sensitive to amoxicillin.

### 3.1. Feed Analyses and Growth Performance

The analyzed nutrient concentration as well as total phenol and condensed tannin contents of all diets are presented in Table 2.

A low supplementation level of GE in the diet (150 g/t; according to supplier’s recommendation) resulted in nearly the same analyzed total phenol content between NC, PC and GE. With a total polyphenol content of GE of ~40% and an inclusion level of 150 g/t, a grape-derived polyphenols supplementation level of 6 mg GAE/100 g FM was calculated. However, the above-mentioned inclusion of GE in the diet influenced the content of condensed tannins in both the starter and the grower diets, and higher amounts were analyzed in the diet (especially in the starter) supplemented with GE.

The results of ADFI, ADG and FCR of all dietary treatments are presented in Table 3.

When observing total trial duration (day 1–day 56), GE had no effect on the growth performance, while PC showed a tendency to improve ADG (*p* = 0.053), compared to NC. On day 13, PC improved piglets’ BW compared to NC and GE (*p* = 0.002). A tendency (*p* = 0.080) was also observed regarding the final BW for PC compared to NC, but not regarding GE.

In the starter phase, PC increased ADFI compared to other treatment groups, and ADG in the early grower phase compared to NC. On day 13, PC improved piglet BW compared to NC and GE (*p =* 0.002). At the end of the trial, piglets from the PC reached higher BW compared to NC (*p* < 0.10). However, neither the GE supplementation nor PC affected the FCR in any phase of the trial.

In the starter phase of the trial, an effect of sex was observed. Female piglets showed an increase in the ADFI in the second week of the trial (*p* = 0.016; interaction sex × week; means not shown). However, male piglets increased FCR in the starter phase (*p* = 0.031).

### 3.2. Apparent Total Tract Digestibility

Uniformly for both sampling points (=overall; tg × T, *p* > 0.05), higher coefficients of ATTD compared to both control groups were observed in GE for most analyzed parameters (DM, OM, gross energy). In addition, GE increased the ATTD of HEE compared to NC, but not PC. However, despite lower values than GE, the PC still increased the ATTD of DM, OM, gross energy and HEE compared to NC (Table 4).

Statistical analysis showed that experimental groups reacted differently between the two sampling points (tg × T, *p* < 0.05) for selected parameters. At T1, but not at T2, there was an observed improvement of the ATTD of Ca for GE, compared with that for NC. GE also improved the ATTD of P at both sampling points compared with treatments NC and PC. However, PC improved the ATTD of P, when compared to NC, but only at T1. Furthermore, a trend for the interaction tg × T (*p* < 0.10) indicated that adding GE increases the ATTD of CP in T1, relative to NC and PC treatments, but this effect was maintained at T2 only in the comparison with NC. Even though statistics indicated interaction in treatment and sampling points for ATTD of ash (tg × T, *p* < 0.05), both PC and GE had increased values compared with that for NC for both T1 and T2.

Overall, there was an effect of sex on the ATTD of DM, OM and gross energy. Female animals had higher digestibility of DM and OM (*p* = 0.004; *p* = 0.013; means not shown) and showed a tendency to increase the digestibility of gross energy (*p* = 0.086; means not shown). Concerning the overall interaction of treatment and sex (*p* < 0.05), male animals in the GE and PC treatments showed increased ATTD of CP compared with the NC group (*p* = 0.041); means not shown). In addition, female animals from GE improved the ATTD of CP, but only when compared with PC.

### 3.3. Microbial Metabolites in Ileal and Colonic Digesta

#### 3.3.1. Concentration of Intestinal SCFA and Lactic Acid (LA)

Table 5 and Table 6 present the effects of dietary treatments on SCFA and LA concentrations in ileal and colonic digesta, respectively.

Digesta samples collected from the ileum did not show any differences in the lactic acid and SCFA concentration at any time point. However, the sum of all SCFA (especially acetic acid) was higher at T2 than T1.

The GE and PC groups showed a tendency (*p* = 0.059) to increase the concentration of lactic acid in the colonic content in comparison with NC when observing T1. There was no effect of any dietary treatment on total SCFA concentrations nor on individual SCFA content. At T2, male animals showed increased concentrations of valeric acid (*p* = 0.010; means not shown).

#### 3.3.2. Concentration of Intestinal Ammonia and Biogenic Amines (BA)

The effects of dietary treatments on ammonia and BA concentrations in ileal and colonic digesta are shown in Table 7 and Table 8.

Overall, concentrations of ammonia and BA in the ileal digesta did not differ between the three treatment groups. At T1, animals fed with GE showed a tendency (*p* = 0.084) for decreased ammonia concentrations. In general, the sum of all BA in ileum was lower in T2 than T1.

Neither ammonia concentrations nor the sum of all BA or individual BA concentrations in the colon were influenced by the dietary treatments in any sampling point of the trial. A tendency for an effect of sex could be observed in T2 for ammonia and total BA, where concentrations were lower for female animals compared with males (*p* = 0.085; *p* = 0.083; means not shown).

## 4. Discussion

Intestinal commensal microbes compete with pathogens for nutrients and binding sites, and they produce metabolites that make important contributions to the regulation of the immune response [48] and in preventing barrier destruction [49]. As mentioned in the Introduction, PWD is commonly associated with *E. coli*. In the present trial, PWD occurred in the first eight days and diarrhea symptoms persisted for the first 3 weeks of the trial, similar to the findings of Amezcua et al. [50]. In the present trial, all analyzed faeces samples from different treatment groups tested positive, inter alia, on ETEC strain K88. Aside from weaning stress including transfer to the trial facilities, blood samples were collected from the piglets two days before weaning (weaning = day 0 of the trial), as well on day 6 of the trial. These analyses are to be published separately. The causes and the consequences of different additional stressors at weaning were well summarized by Martínez-Miró et al. [51]. Weaning stressors may have contributed to increased susceptibility for developing diarrhea in this study, even though the piglets were not deliberately challenged with any pathogenic agent. Despite antibiotic treatment in the PC group during the first five days of the trial, symptoms of diarrhea were present in all treatment groups in the first 3 weeks. However, according to the results of the weaning performance in the starter phase, animals in the PC treatment group recovered more quickly from the described challenges at the beginning of the trial.

In this feeding trial, there was no considerable interaction between the experimental factors of sex and dietary treatments. The few interactions observed did not show directed trends. For this reason, focus is placed on discussing the main effect of treatment as well as the interaction of treatment and sampling point.

### 4.1. Performance

Poor performance is associated with reduced and variable feed intake and can occur subsequent to weaning [52]; consequently, there is a need for dietary supplements to alleviate weaning stress and thereby improve performance. Addition of certain amounts of grape polyphenols in the diet has been shown previously to have beneficial effects on piglet performance [53]. In the present trial, however, dietary supplementation with GE, compared with NC and PC treatment groups, showed no effect on ADG, ADFI and FCR during the entire trial. Fiesel et al. [34] obtained similar results regarding ADG and ADFI by feeding grape seed and grape marc meal extract to growing pigs. However, their results were achieved with a supplementation level of grape polyphenols at 85 mg GAE/100 g FM, considerably higher than the grape polyphenol supplementation level of 6 mg GAE/100 g FM in the present trial. In the above cited study [34], there was a tendency towards an improved FCR. In an earlier study, the same group [54] observed an improvement of FCR in growing piglets that received a similar grape product. In a piglet trial with different polyphenol sources, grape seed extract (grape-derived polyphenol supplementation level was not stated) showed no effect on the performance of the piglets challenged with ETEC, although this product was shown to inhibit toxin production of ETEC in an in vitro trial within the same study [55]. Broiler studies in which different diets were supplemented with polyphenols from grape seed extract [32] and grape pomace (grape-derived polyphenol supplementation level was not stated) [56] reported no effect on animal performance. Conversely, a growth depression was manifested [31] when supplementing with a very high concentration of polyphenols from grape, i.e., 4670 mg GAE/100 g FM (compared with only 6 mg GAE/100 g FM in the present trial). In addition to differences in composition and concentration of grape polyphenols, other factors play a role in affecting the differences in these findings, including onset and duration of the dietary supplementation of polyphenols (polyphenol supplementation in the diet before the weaning) and different study designs [53].

In the present trial, PC increased the ADFI during the starter phase (day 1–day 13). This effect occurred in the second week post weaning or, more precisely, in the week after the antibiotic treatment with amoxicillin. However, this trend was evident only numerically (*p* < 0.05) during the rest of the trial and there was no effect of PC on ADFI after 8 weeks of the trial. One explanation could be the presence of diarrhea symptoms (as described above) from the beginning of the trial. Jensen et al. [57] demonstrated that increased gut flow during diarrhea can reduce the absorption of orally administered amoxicillin to the weaning piglets infected with *E. coli*. However, during PWD, antibiotics are often administered orally (via feed or drinking water) since single-animal parenteral therapy involves higher cost, management and time demands. Nonetheless, the higher feed intake during the most challenging time of weaning led to benefits in BW gain in the subsequent phases: increased ADG during the early grower phase (day 14–day 24), compared with piglets fed NC, resulted in the tendency (*p* = 0.053) of PC to improve ADG for the whole trial duration (day 1–day 56). This shows that antibiotic treatment in the first week of the trial had carry-over effects, which appeared in the weeks that followed. However, this effect was not observed in the later growing phase. This is consistent with findings in another study [58], where three different antibiotic treatment groups (including amoxicillin) showed an improvement of ADG and ADFI of the weaning piglets during or following the antibiotic treatment, but this advantage disappeared one week after the antibiotic treatment. However, to be able to discuss further the impact duration of the five days antibiotic exposure in the present trial, additional information and analyses would be necessary.

In terms of performance, GE in the diet of weaning piglets did not improve parameters on the same level as PC. However, the final BW of the piglets fed GE lay between those for NC and PC, thus differing statistically from neither PC nor NC. Despite the recorded superior effects of PC compared to GE on performance, it should be stated that repeated use of antibiotics for a treatment of PWD increases the risk of antibiotic resistance development in the gut microbiota, such as ETEC [50]. This suggests that the impact of the antibiotic treatment, as used in the present trial, is presumed to have a limited duration. The absence of greater effects by GE supplementation, when compared to NC or PC treatments, needs to be considered with regard to the inclusion level of grape polyphenols. In the present trial, the GE supplementation dosage might have been set too low in terms of its effect on performance. The supplementation of polyphenols derived from GE did not exhibit differences in the analysis of total phenol content of the diets (Table 2), partly due to the limited specificity of this traditional method.

### 4.2. Apparent Total Tract Digestibility

Besides poor performance, changes in the structure of the gastrointestinal tract and decreased absorptive capacity of the small intestine post weaning have been observed in other studies [52,59]. As already pointed out by Jensen [60], any change in the metabolic demand and microbial load especially in the small intestine, has a great impact on the health of the host. Additionally, the physiology of the gastrointestinal tract of the weaning piglet (still low activity of digestive enzyme due to immature stomach and pancreatic function) plays a notable role in the lower digestibility [61].

The supplementation of GE in the diet of weaning piglets clearly improved the ATTD of nutrients in the present trial (DM, OM, ash, gross energy, CP, HEE, P; Table 4). In a study with fattening pigs [33], potential positive effects on the digestibility in diets supplemented with 186.3 mg GAE/100 g FM grape pomace polyphenols (DM and nitrogen) were suggested. This is a considerably higher grape polyphenol supplementation, compared with the 6 mg GAE/100 g FM supplementation level in the present trial. In a feeding trial in which grape seed and grape marc meal extract was fed to piglets [34], investigations were made of the ATTD of CP, crude fat, crude fibre and N-free extracts. With grape-derived polyphenols supplementation of 85 mg GAE/100 g FM, they obtained positive results only regarding crude fat digestibility. The effect of polyphenols in grape pomace was also studied by others [56,62], and in both studies the authors reported no changes in ATTD of CP in broilers fed with grape pomace. Polyphenol supplementation derived from grape in these studies were again at much higher amounts than in the present study, up to 150 mg GAE/100 g FM. However, especially the tannin part of polyphenols needs to be considered regarding ATTD. Different studies were performed to explore the impact of tannins on protein digestibility, because tannins interact with proteins, including (digestive) enzymes in the gut lumen, which depends on the concentration of tannins and intestinal conditions, such as pH value [6]. In a study with different concentrations of tannins in diets of growing pigs containing sorghum there were some negative effects on protein digestibility [63]. This result was reached at the highest tannin content in the diet (1000 mg condensed tannins/100g DM). Comparatively, in the present trial with 7 mg condensed tannins/100 g DM (Table 2), the concentration of GE seemed to have been appropriately selected, considering the CP digestibility, because no impairment was observed.

Besides having a positive effect compared to NC, the GE treatment improved the ATTD of all nutrients, even exceeding the level of the PC, with exception of ATTD of ash and HEE. However, PC showed positive effects on the ATTD of DM, OM, ash, gross energy, HEE and P, compared to NC.

Concerning the duration of the impact, the improvement of ATTD of GE compared to NC could be observed for most nutrients (except for ATTD of Ca) at both sampling points. In contrast, the PC group showed better results in younger animals (4 weeks post weaning; 3 weeks after antibiotic administration) compared to NC but lost this advantage in older animals (8 weeks post weaning; 7 weeks after antibiotic administration), as observed for ATTD of DM and P (Table 4). In general, antibiotic treatment is expected to be more effective at a critical stage of life (weaning, younger animals) and the effect of the therapy is more noticeable in sick animals and animals housed under conditions of poor hygiene [64,65]. This is an additional reason why antibiotic application to prevent performance losses plays such an important role at weaning and why the effect of antibiotic treatment—also in the present trial—was particularly visible for the sampling at 4 weeks post weaning.

The ability of tannins in protein binding could be important to inactivate some proteins involved in ETEC pathogenesis, and tannins may also bind the substrate required for microbial growth and make it unavailable [6,66]. In higher concentrations, tannins can inhibit the adhesion of pathogens to the intestinal epithelium, which is the prerequisite for a biofilm formation and a subsequent toxin secretion [66,67]. When grape polyphenols were used, a dosage-dependent inhibition of biofilm formation of *E. coli* was described [28]. This reduces the number of pathogens and their negative impact on gastrointestinal tract, as well as pathogenic utilization of nutrients and energy loss due to microbial fermentation, improving immune response and the absorption of nutrients [6]. Such mechanisms of tannins may explain the positive effect of GE treatment in ATTD parameters.

Different antibiotics exert bacterial growth-inhibiting impact. The absence of effectiveness of antibiotics against some bacteria was related exactly to the formation of the bacterial biofilm. The interaction of antibiotics with the microbiome, which is the main but not the only effect in the gut, can indirectly affect the intestinal epithelial barrier, because microbiota modulate the thickness of the protective intestinal mucus [68]. Antibiotics can also directly affect the intestinal epithelium [69], by compromising the epithelial gene expression, which can be reflected in alterations in the gut-associated immune system [70]. Nevertheless, the changes in microbial load in favor of beneficial bacteria is probably the mode of action of antibiotics to increase nutrient absorption [49].

In summary, GE supplementation improved the ATTD above the level of that of PC. Based on these findings, a positive effect on the performance of the weaning piglets was expected regarding the GE supplementation, but this could not be confirmed in the present trial. These results are consistent with those of Brenes et al. [32], who observed that a polyphenol supplementation from grape seed extract at a level of 342 mg GAE/100 g FM in the broiler diet, increased the ileal protein digestibility after 3 weeks of the trial. However, this effect on ileal protein digestibility disappeared 3 weeks later and showed no effect on broiler performance. Similarly, in the present trial, piglets fed GE showed greater ATTD of CP and Ca at T1, but this effect disappeared 4 weeks later. However, the ATTD of DM and P, which was improved compared to NC at T1, was also increased compared to PC at T2. In the present trial, the dietary supplementation of polyphenols from grape was much lower, at 6 mg GAE/100 g FM, than in the aforementioned study, which probably caused the variations in the digestibility in older animals between these two studies. Although the transferability of the positive effects of ATTD on improved performance was not observed for piglets in the present trial, one can hypothesize that longer feeding duration of GE (additionally in fattening period) and possibly higher polyphenol inclusion would be necessary to show a positive effect of GE supplementation.

In the present trial, ATTD of P is higher than values previously reported [71,72,73] in pigs. However, our findings are comparable to observations of Zhang and Adeola [74] for ATTD of P in growing pigs. In general, in our study, the coefficients of ATTD of nutrients were quite high. This may have been affected in part by the relatively low TiO_2_ concentrations analyzed in feed (NC = 0.17%; PC = 0.16%; GE = 0.17% as fed), compared to the concentrations of TiO_2_ applied to the diets (0.30%).

### 4.3. Microbial Metabolites

Gut microbiota and its metabolites may influence the development of PWD and the health of the host [75]. This occurs by preventing pathogenic bacteria from colonizing the gastrointestinal tract [68]. Beneficial bacteria such as lactic acid producing bacteria (e.g., lactobacilli) can support commensal gut bacteria and decrease the level of diarrhea-causing pathogens and improve the intestinal microbial balance. Antibiotic treatment and PFA are reported to both have effects on the gut microbiome [76], but the mechanisms may differ.

Diets containing polyphenols (especially the tannin part) in specific amounts can modify intestinal microbiota by promoting the growth of lactic acid producing bacteria [29]. Tannins exert this function probably by acting as substrate for the lactic acid-producing bacteria [77], as some species belonging to the group of lactic acid bacteria contain tannase [78]. Tannins therefore may inhibit the growth of pathogenic bacteria, but not lactic acid-producing bacteria. A reason might be the ion chelation (minerals such as iron and zinc required for bacterial growth) induced by tannins which obviously does not play a role for lactic acid producing bacteria [6]. Conversely, a broad-spectrum antibiotic may impact not only pathogenic, but also the commensal bacteria, although penicillins (e.g., amoxicillin) seem to have very low impact on microbiota in general [69]. Besides the host-commensal bacteria-pathogens interaction, the diet composition (protein and carbohydrate content), as well as naturally occurring compounds such as polyphenols, play a role for the gut microbiota eubiosis [79] and microbial metabolites (e.g., SCFA, microbial metabolites of non-absorbed proteins). Due to an increased digestibility and, subsequently, reduced amount of undigested dietary protein flowing into the large intestine, decreased proliferation of potential pathogenic bacteria and their metabolites (e.g., ammonia, BA) can be assumed.

Although differences between the treatment groups PC and GE were observed for ATTD, the concentration of microbial metabolites did not differ. In colon digesta, we determined the tendency (*p* = 0.059) of GE and PC to improve LA concentrations in the 4 weeks post weaning (Table 6). This confirms that modulation of the gut microbiota is more pronounced in the first weeks post weaning, with increasing beneficial lactic acid bacteria. In general, microbial activity is expected to increase and be more stable with the age of the animals [60]; this corresponds with our findings for the late sampling point, regarding LA concentrations and sum of all SCFA.

Some dietary carbohydrates can pass to the large intestine undigested, being fermented by colonic anaerobic bacteria and producing microbial metabolites such as short-chain fatty acids (SCFA). Acetic, propionic and butyric acid are the most abundant SCFA, which are rapidly absorbed and metabolized in, e.g., enterocytes or the liver [80]. Therefore, SCFA play an important role for health and energy metabolism of the host [81]. A potential beneficial impact of GE treatment, i.e., an increased concentration of propionic and butyric acid in colonic digesta was reported [82] in a study with weaning piglets (grape-derived polyphenol supplementation level not specified). Even more considerable effects were found in ileal content, where propionic and butyric acid concentrations were increased in the grape polyphenol group over the level of AGP [82]. Han et al. [83] also reported increased SCFA concentrations in the digesta collected from the ileum and colon in a trial with weaning piglets fed grape seed compared to NC and AGP. Unfortunately, also in a study by Han et al. [83], the level of grape-derived polyphenol supplementation was not specified. These results, however, need to be evaluated cautiously in comparison with the results from the present trial, since the dosage and duration of the supplementation of AGP in Han et al. [83] differed from that of the therapeutic antibiotic treatment (PC treatment) in the present trial. In the present trial, no difference was observed in the concentrations of SCFA among the treatments at both sampling points (T1 and T2). This is surprising, considering the changes in LA concentration (the tendency at T1; *p* = 0.059) found in colon (Table 6), as the intestinal microflora can utilize LA as a precursor for SCFA production [84]. In addition, PC did not show an influence on colonic SCFA concentration in the present trial either, although a study with rats reported that intramuscular amoxicillin treatment for 14 days can suppress SCFA production and excretion [85]. This difference may be attributed to the mode of application and duration of the antibiotic treatment, suggesting that the effect of the therapeutic amoxicillin treatment may already have disappeared by the sampling time.

Undigested dietary protein passing through the small intestine reaches the large intestine and is used as substrate for microorganisms. Not only the presence of undigested dietary protein, but also endogenous protein secretion and the immature stage of the gut at weaning (incomplete development of enzymatic activity) may favour bacterial fermentative activity in the large intestine. In consequence, products of microbial protein metabolism such as ammonia and BA, increase the risk of diarrhea [86]. Improved CP digestibility, as observed in the present trial by dietary inclusion of GE, serves as the best prerequisite for decreasing concentrations of ammonia and BA in the digesta (ileum and proximal colon; Table 7 and Table 8). However, in the present trial, the digesta samples obtained from the ileum and colon did not respond to the GE or antibiotic supplementation in the diet regarding BA concentrations. There was a trend for reduced ileal ammonia concentrations in GE group at T1, while no effect was observed on ammonia concentration in colonic digesta samples. In a study with piglets [87], the addition to the diet of polyphenols derived from chestnut wood (polyphenol contents not stated; 79–315 mg tannin supplementation/100 g FM) reduced caecal ammonia content. Furthermore, Colombino et al. [88] reported a decrease in ammonia concentration in the ileum and caeca of broilers fed different polyphenol-containing diets with grape-derived polyphenol supplementation of 80–174 mg GAE/100 g FM. However, this supplementation level is much greater than the supplementation level in the present trial of 6 mg GAE/100 g FM. Although ATTD was improved, we assume that the lower concentration of supplemented polyphenols from grape in the present trial was not sufficient to alter the BA and ammonia concentration in ileal and colonic digesta. However, the contribution of ileal endogenous protein losses may be affected by a specific dietary intervention [89], therefore further influencing the calculation of ATTD of CP. This may have also played a role in the present trial, potentially changing the bacterial substrate availability and, subsequently, the concentration of protein-derived microbial metabolites.

Regarding the possible mode of action of GE and PC, compared to the NC treatment, when observed for the whole trial duration, the impact of GE and PC treatments seems to be similar in relation to growth performance, ATTD and microbial metabolites. Potential difference in the mechanism was observed following the weaning, where PC showed initial advantages regarding the piglet performance. In addition, GE showed substantial impact on ATTD of nutrients compared to PC. Neither the PC nor GE treatments had the ability to modify the overall microbial metabolites in the ileum and colon. Overall, the comparability of the results achieved in this study and previous scientific findings appeared to be limited. One reason may be that the detailed information on the composition and supplementation level of grape-derived polyphenols is frequently missing in previous studies. In addition, studies with an antibiotic treatment as positive control mostly used in-feed AGP compared to the five days antibiotic treatment that was used in the present trial.

## 5. Conclusions

The PC group showed positive effects on the piglet performance (2 weeks post weaning); however, this occurred without longer lasting effects at the end of the trial (8 weeks post weaning). Neither PC nor GE was found to markedly improve total growth performance (day 1–day 56) compared to NC. For ATTD of nutrients, the response to GE was higher than to PC. However, this effect was not directly correlated to microbial metabolites concentrations. Using NC and PC was successful to obtain additional insights into the mode of action of both treatments. Our supposition is that continuous inclusion of grape extract at the selected dosage could be used as an equivalent to the frequently pro- or metaphylactically applied antibiotic treatment in the diets of weaning piglets without negative effect on the performance.

## Figures and Tables

**Table 1:** Composition of the experimental diets (as-fed basis) in the starter and grower phases.

Ingredient (%)	Starter (Day 1–Day 13)	Grower (Day 14–Day 56)
Wheat (winter)	23.04	26.50
Maize	18.00	20.00
Maize (extruded)	9.50	-
Barley	20.00	30.00
Soybean meal without hulls	9.00	18.10
Soy protein concentrate	5.00	-
Vegetable oil	0.50	0.60
Potato protein	2.50	-
Lactose	2.50	-
Whey powder	6.00	-
L-lysine (HCl)	0.61	0.55
DL- methionine	0.29	0.27
L-threonine	0.25	0.32
L-tryptophan	0.07	0.04
Monocalcium phosphate	0.75	1.00
Calcium carbonate	0.88	1.10
Salt	0.31	0.42
Titanium dioxide	-	0.30
Vitamin and trace element premix ^1^	0.30	0.30
Maize premix, ground ^2^	0.50	0.50
Chemical composition		
ME (MJ/kg) ^3^	14.00	13.40
Crude protein (%)	18.10	17.90
Crude fibre (%)	2.70	3.30
Starch (%)	43.90	45.40
Lysine (%)	1.31	1.20
Methionine and cysteine (%)	0.80	0.80
Threonine (%)	0.88	0.92
Tryptophan (%)	0.27	0.24
Calcium (%)	0.70	0.81
Phosphorus (%)	0.53	0.59
Sodium (%)	0.20	0.20
SID lysine (%)	1.17	1.05
SID methionine and cysteine (%)	0.68	0.67
SID threonine (%)	0.74	0.75
SID tryptophan (%)	0.23	0.20

SID = standardized ileal digestibility; ^1^ Supplied per kg of diet: 10,000 IU vitamin A, 2000 IU vitamin D_3_, 40 mg vitamin E, 1.5 mg vitamin K_3_, 1.0 mg vitamin B_1_, 4.0 mg vitamin B_2_, 1.5 mg vitamin B_6_, 0.020 mg vitamin B_12_, 30 mg nicotinic acid (niacin), 15 mg D-pantothenic acid, 150 mg choline chloride, 0.05 mg biotin, 0.4 mg folic acid, 100 mg Fe, 20 mg Cu, 70 mg Zn, 30 mg Mn, 0.70 mg I, 0.25 mg Se; ^2^ Consisting of experimental premix mixture containing 100% ground maize for NC and PC, or PFA in GE (3% grape extract, 97% ground maize). ^3^ ME = metabolizable energy; calculated based on the equation published by GfE [39].

**Table 2:** Analyzed nutrient concentration in all diets (as-fed) in starter and grower phases.

	Starter (Day 1–Day 13)			Grower (Day 14–Day 56)		
Item ^1^	NC	PC	GE	NC	PC	GE
Analysed composition						
Dry matter (%)	89.53	89.74	89.88	88.90	88.96	89.17
Ash (%)	5.48	5.49	6.34	5.04	5.71	5.25
Crude protein (%)	18.72	18.96	19.85	17.62	17.07	17.97
Hydrolyzed ether extract (%)	3.94	3.94	4.16	3.85	3.86	3.76
Starch (%)	43.37	42.89	41.29	47.02	46.08	45.62
Sugar (%)	6.50	6.49	6.79	3.63	3.54	3.49
Crude fibre (%)	2.15	2.52	2.01	2.57	2.87	2.34
ME (MJ/kg) ^2^	14.3	14.2	14.3	14.1	13.8	14.1
Ca (%)	0.99	0.99	1.13	0.63	0.67	0.59
P (%)	0.57	0.55	0.56	0.60	0.58	0.62
Total phenols ^3^, g GAE/100 g ^4^ DM	0.41	0.38	0.38	0.34	0.34	0.34
Condensed tannins, mg CT/100 g DM	4.82	4.78	7.06	8.17	7.07	8.87

^1^ NC = negative control; PC = positive control, NC + 20 mg amoxicillin/kg body weight twice a day for the first 5 days of the feeding trial; GE = NC + grape extract, 150 g/t; DM = dry matter. ^2^ ME = metabolizable energy; calculated based on the equation published by GfE [39]. ^3^ total phenol contents of entire diets including native concentrations of feed. ^4^ GAE = Gallic acid equivalents as measured by Folin–Ciocalteau method.

**Table 3:** Effect of using NC and PC for testing dietary GE supplementation on average daily feed intake (ADFI), average daily gain (ADG) and feed conversion rate (FCR) of weaning piglets ^1^.

Item	Treatment Group (tg) ^2^			Sex		SEM ^3^	*p*-Value			
	NC	PC	GE	m	w		tg	Sex	tg × Week	tg × Sex
BW (kg/piglet)										
day 0	6.89	6.91	6.90	6.97	6.83	0.1	0.99	0.17		0.99
day 13	7.87 ^b^	8.50 ^a^	8.02 ^b^	8.17	8.09	0.1	0.002	0.57		0.52
day 24	10.1	14.0	10.7	10.7	12.5	1.6	0.17	0.30		0.47
day 56	23.8 ^(b)^	26.3 ^(a)^	25.4 ^(ab)^	24.8	25.5	0.8	0.080	0.48		0.48
ADFI (g/day/piglet)										
Starter (day 1–day 13)	107 ^b^	134 ^a^	102 ^b^	112	117	4.4	<0.001	0.36	0.020	0.93
Early grower (day 14–day 24)	333	383	353	351	361	17	0.12	0.61	0.003	0.89
Late grower (day 25–day 56)	863	943	905	904	903	32	0.21	0.98	<0.001	0.55
Total (day 1–day 56)	541	601	566	569	571	19	0.11	0.89	0.31	0.64
ADG (g/day/piglet)										
Starter (day 1–day 13)	116	134	124	123	124	8.6	0.18	0.91	0.55	0.68
Early grower (day 14–day 24)	234 ^b^	296 ^a^	270 ^ab^	262	271	14	0.006	0.60	0.075	0.72
Late grower (day 25–day 56)	530	578	558	554	556	20	0.23	0.94	0.46	0.47
Total (day 1–day 56)	348 ^(b)^	388 ^(a)^	373 ^(ab)^	371	375	12	0.05	0.77	0.47	0.52
FCR (g/g)										
Starter (day 1–day 13)	1.12	1.07	1.21	1.25	1.04	0.08	0.62	0.031	0.083	0.16
Early grower (day 14–day 24)	1.50	1.38	1.36	1.43	1.40	0.05	0.10	0.65	0.55	0.28
Late grower (day 25–day 56)	1.62	1.64	1.62	1.62	1.63	0.02	0.76	0.48	0.48	0.34
Total (day 1–day 56)	1.47	1.42	1.46	1.48	1.42	0.02	0.77	0.054	0.030	0.40

^1^*n* = 12 pens/tg; 5 animals/pen on day 27/28, 4 animals/pen on day 55/56; ^2^ NC = negative control; PC = positive control, NC + 20 mg amoxicillin/kg body weight twice a day for the first 5 days of the feeding trial; GE = NC + grape extract, 150 g/t. ^3^ SEM Standard error of mean based on LSMeans. ^a,b^ Values within a row without a common superscript differ at *p* ≤ 0.05 (values within brackets *p* < 0.10).

**Table 4:** Effect of using NC and PC for testing dietary GE supplementation on apparent total tract digestibility of weaning piglets ^1^.

Item ^4^	Treatment Group (tg) ^2^			Sex		SEM ^3^	*p*-Value				
	NC	PC	GE	m	f		tg	Sex	T	tg × Sex	tg × T
DM (%)											
Overall	86.3 ^c^	87.3 ^b^	88.3 ^a^	86.9	87.7	0.2	<0.001	0.004	<0.001	0.073	0.31
T1	85.2 ^b^	86.7 ^a^	87.6 ^a^	86.1	87.0	0.3	<0.001	0.029		0.23	
T2	87.3 ^b^	88.0 ^b^	88.9 ^a^	87.8	88.4	0.3	0.001	0.068		0.30	
OM (%)											
Overall	88.3 ^c^	89.2 ^b^	90.0 ^a^	88.9	89.5	0.2	<0.001	0.013	<0.001	0.087	0.58
T1	87.4 ^b^	88.5 ^ab^	89.4 ^a^	88.1	88.8	0.3	0.001	0.064		0.29	
T2	89.2 ^b^	89.7 ^ab^	90.7 ^a^	89.6	90.1	0.3	0.003	0.10		0.30	
Ash (%)											
Overall	66.0 ^b^	71.5 ^a^	72.2 ^a^	69.9	69.9	0.4	<0.001	0.95	0.90	0.56	0.008
T1	65.0 ^b^	71.6 ^a^	73.0 ^a^	69.8	70.7	0.7	<0.001	0.76		0.80	
T2	67.1 ^b^	71.5 ^a^	71.3 ^a^	70.0	69.9	0.5	<0.001	0.74		0.66	
Gross energy (%)											
Overall	85.1 ^c^	86.2 ^b^	87.5 ^a^	86.0	86.6	0.3	<0.001	0.086	<0.001	0.08	0.30
T1	83.6 ^b^	85.1 ^ab^	86.6 ^a^	84.8	85.4	0.5	0.001	0.34		0.23	
T2	86.7 ^b^	87.4 ^ab^	88.4 ^a^	87.2	87.8	0.3	0.002	0.10		0.32	
CP (%)											
Overall	82.9 ^b^	84.0 ^b^	86.3 ^a^	84.0	84.7	0.4	<0.001	0.19	<0.001	0.041	0.080
T1	81.1 ^b^	82.6 ^b^	85.9 ^a^	82.9	83.5	0.7	<0.001	0.50		0.20	
T2	84.7 ^b^	85.3 ^ab^	86.6 ^a^	85.2	85.9	0.5	0.022	0.19		0.19	
HEE (%)											
Overall	57.0 ^b^	60.9 ^a^	64.3 ^a^	60.2	61.3	1.0	<0.001	0.34	<0.001	0.18	0.12
T1	50.0 ^b^	55.8 ^ab^	60.2 ^a^	55.1	55.6	1.8	0.002	0.84		0.20	
T2	64.1 ^b^	65.9 ^ab^	68.4 ^a^	65.2	67.1	1.0	0.019	0.11		0.22	
Ca (%)											
Overall	72.4	73.6	75.0	74.0	73.4	1.1	0.23	0.66	<0.001	0.36	0.027
T1	76.4 ^b^	80.8 ^ab^	83.0 ^a^	80.0	80.1	1.4	0.008	0.98		0.34	
T2	68.4	66.5	67.1	67.9	66.7	1.6	0.69	0.54		0.69	
P (%)											
Overall	83.9 ^c^	85.3 ^b^	87.8 ^a^	85.6	85.7	0.4	<0.001	0.95	<0.001	0.87	0.002
T1	81.4 ^c^	84.1 ^b^	87.2 ^a^	84.4	84.1	0.7	<0.001	0.72		0.98	
T2	86.4 ^b^	86.5 ^b^	88.3 ^a^	86.9	87.2	0.2	<0.001	0.26		0.15	

^1^*n* = 1 pooled sample/pen; 12 pens/tg; DM = dry matter; OM = organic matter; CP = crude protein; HEE = acid-hydrolyzed ether extract. ^2^ NC = negative control; PC = positive control, NC + 20 mg amoxicillin/kg body weight twice a day for the first 5 days of the feeding trial; GE = NC + grape extract, 150 g/t. ^3^ SEM Standard error of mean based on LSMeans. ^4^ T (sampling point): T1, day 25/26; T2, day 53/54, overall (T1 + T2). ^a,b,c^ Values within a row without a common superscript differ at *p* ≤ 0.05.

**Table 5:** Effect of using NC and PC for testing dietary GE supplementation on the concentrations of SCFA (mmol/kg) and lactic acid (mmol/kg) in ileal content of weaning piglets in two different sampling points ^1^.

Item ^4^	Treatment Group (tg) ^2^			Sex		SEM ^3^	*p*-Value				
	NC	PC	GE	m	f		tg	Sex	T	tg × Sex	tg × T
Total SCFA											
Overall	22.6	22.6	21.0	22.8	21.3	1.9	0.80	0.50	<0.001	0.72	0.63
T1	15.4	17.7	14.0	16.5	14.9	2.4	0.55	0.56		0.65	
T2	29.8	27.4	28.1	29.1	27.7	3.0	0.85	0.70		0.50	
Acetic acid											
Overall	16.1	15.3	14.3	15.8	14.6	1.7	0.74	0.53	<0.001	0.76	0.84
T1	10.4	10.9	8.6	10.7	9.24	1.7	0.62	0.46		0.75	
T2	21.8	19.8	19.9	20.9	20.0	2.9	0.86	0.78		0.58	
Propionic acid											
Overall	4.04	4.60	4.04	4.42	4.02	0.4	0.57	0.42	0.27	0.99	0.27
T1	3.54	4.88	3.42	4.23	3.66	0.8	0.37	0.55		0.91	
T2	4.53	4.31	4.65	4.61	4.38	0.3	0.68	0.48		0.45	
Valeric acid											
Overall	1.78	2.05	2.06	1.91	2.02	0.2	0.43	0.56	0.46	0.025	0.75
T1	1.76	2.16	2.20	1.91	2.18	0.3	0.56	0.46		0.049	
T2	1.82	1.95	1.92	1.92	1.87	0.2	0.82	0.78		0.15	
Heptanoic acid											
Overall	-	-	-	-	-	-	-	-	-	-	-
T1	n.d.	n.d.	n.d.	n.d.	n.d.	-	-	-		-	
T2	1.66	1.59	1.62	1.62	1.63	0.1	0.99	0.99		0.49	
Lactic acid											
Overall	42.3	44.6	41.6	42.4	43.4	6.2	0.94	0.89	0.83	0.50	0.59
T1	36.5	45.3	44.5	41.2	43.0	6.7	0.59	0.81		0.36	
T2	46.8	44.0	38.8	43.6	43.7	11	0.82	0.99		0.88	

^1^*n* = 1 animal/pen; 12 pens/tg; n.d. = not detectable; SCFA (isocapronic and hexanoic acid) beneath the detection level not shown; ^2^ NC = negative control; PC = positive control, NC + 20 mg amoxicillin/kg body weight twice a day for the first 5 days of the feeding trial; 150 g/t; GE = NC + grape extract, 150 g/t. ^3^ SEM Standard error of mean based on LSMeans. ^4^ T (sampling point): T1, day 27/28; T2, day 55/56, overall (T1 + T2).

**Table 6:** Effect of using NC and PC for testing dietary GE supplementation on the concentrations of SCFA (mmol/kg) and lactic acid (mmol/kg) in colonic content of weaning piglets in two different sampling points ^1^.

Item ^4^	Treatment Group (tg) ^2^			Sex		SEM ^3^	*p*-Value				
	NC	PC	GE	m	f		tg	Sex	T	tg × Sex	tg × T
Total SCFA											
Overall	194	189	177	189	188	8.4	0.33	0.93	0.001	0.82	0.50
T1	178	165	167	171	172	11	0.65	0.92		0.50	
T2	210	213	187	204	203	11	0.22	0.91		0.98	
Acetic acid											
Overall	106	101	95.8	99.1	103	4.3	0.32	0.45	0.001	0.93	0.58
T1	99.0	89.3	89.1	92.9	92.0	5.3	0.34	0.88		0.41	
T2	112	114	103	105	114	6.8	0.49	0.28		0.84	
Propionic acid											
Overall	51.5	48.2	45.0	49.7	46.9	2.4	0.21	0.34	<0.001	0.61	0.56
T1	44.4	41.3	41.5	42.9	41.9	3.0	0.71	0.79		0.76	
T2	58.7	55.1	48.5	56.3	51.9	4.0	0.23	0.34		0.55	
Isobutyric acid											
Overall	3.41	3.25	3.29	3.17	3.47	0.3	0.91	0.38	0.066	0.60	0.50
T1	3.31	2.68	3.03	2.72	3.30	0.4	0.52	0.20		0.34	
T2	3.52	3.82	3.55	3.62	3.64	0.4	0.81	0.97		0.99	
Butyric acid											
Overall	23.9	26.2	23.6	25.1	24.1	1.7	0.50	0.62	0.038	0.40	0.49
T1	21.7	22.6	23.0	22.0	22.9	2.3	0.92	0.73		0.30	
T2	26.0	29.7	24.7	28.1	25.2	2.0	0.18	0.26		0.45	
Isovaleric acid											
Overall	1.85	1.82	1.81	1.64	2.01	0.2	0.99	0.18	0.67	0.47	0.78
T1	1.98	1.75	1.91	1.62	2.15	0.3	0.89	0.17		0.44	
T2	1.72	1.89	1.69	1.67	1.87	0.2	0.73	0.42		0.75	
Valeric acid											
Overall	8.02	8.18	7.18	8.25	7.33	0.8	0.67	0.34	0.90	0.38	0.54
T1	7.59	7.71	7.88	7.23	8.22	1.2	0.99	0.47		0.31	
T2	8.43	8.66	6.44	9.24	6.45	0.8	0.17	0.01		0.12	
Lactic acid											
Overall	1.25	1.35	1.40	1.27	1.40	0.1	0.62	0.28	0.18	0.90	0.064
T1	1.13 ^(b)^	1.57 ^(a)^	1.56 ^(a)^	1.40	1.44	0.1	0.059	0.78		0.52	
T2	1.38	1.13	1.25	1.14	1.36	0.2	0.56	0.24		0.33	

^1^*n* = 1 animal/pen; 12 pens/tg; SCFA (isocapronic and hexanoic acid) beneath the detection level not shown; ^2^ NC = negative control; PC = positive control, NC + 20 mg amoxicillin/kg BW twice a day for the first 5 days of the feeding trial; 150 g/t; GE = NC + PFA grape extract, 150 g/t. ^3^ SEM Standard error of mean based on LSMeans. ^4^ T (sampling point): T1, day 27/28; T2, day 55/56, overall (T1 + T2). ^(a,b)^ Values within a row without a common superscript differ at *p* ≤ 0.10.

**Table 7:** Effect of using NC and PC for testing dietary GE supplementation on the concentrations of ammonia (µg/g) and biogenic amines (BA) (mmol/kg) in ileal content of weaning piglets in two different sampling points ^1^.

Item ^4^	Treatment Group (tg) ^2^			Sex		SEM ^3^	*p*-Value				
	NC	PC	GE	m	f		tg	Sex	T	tg × Sex	tg × T
Ammonia											
Overall	179	165	169	172	170	8.0	0.47	0.87	0.055	0.52	0.11
T1	180 ^(a)^	159 ^(ab)^	147 ^(b)^	163	161	10	0.084	0.85		0.95	
T2	177	171	192	180	180	12	0.48	0.96		0.45	
Total BA											
Overall	4.02	4.48	4.38	4.34	4.24	0.5	0.79	0.87	0.039	0.88	0.77
T1	4.33	5.13	5.21	4.85	4.93	0.8	0.67	0.93		0.21	
T2	3.69	3.83	3.54	3.82	3.56	0.6	0.95	0.72		0.29	
Ethanolamine											
Overall	0.26	0.22	0.26	0.26	0.23	0.04	0.78	0.54	0.72	0.61	0.71
T1	0.28	0.20	0.24	0.26	0.22	0.04	0.36	0.38		0.53	
T2	0.24	0.25	0.28	0.27	0.25	0.08	0.94	0.83		0.27	
Putrescin											
Overall	0.32	0.38	0.40	0.38	0.36	0.07	0.69	0.73	0.001	0.95	0.37
T1	0.39	0.54	0.59	0.53	0.49	0.1	0.46	0.79		0.54	
T2	0.26	0.22	0.21	0.24	0.22	0.06	0.82	0.81		0.24	
Cadaverin											
Overall	2.05	2.48	2.21	2.22	2.27	0.4	0.73	0.92	0.010	0.95	0.70
T1	2.45	3.19	2.89	2.73	3.00	0.6	0.68	0.75		0.35	
T2	1.66	1.77	1.52	1.72	1.59	0.5	0.94	0.83		0.33	
Histamine											
Overall	0.38	0.50	0.59	0.53	0.45	0.1	0.39	0.55	0.71	0.75	0.55
T1	0.39	0.45	0.70	0.55	0.47	0.2	0.43	0.70		0.56	
T2	0.36	0.55	0.48	0.50	0.43	0.1	0.59	0.63		0.50	
Spermidine											
Overall	0.51	0.49	0.48	0.51	0.48	0.04	0.90	0.62	0.007	0.066	0.98
T1	0.43	0.42	0.41	0.43	0.41	0.05	0.97	0.71		0.041	
T2	0.58	0.57	0.54	0.58	0.55	0.07	0.92	0.73		0.40	
Spermine											
Overall	0.48	0.48	0.49	0.49	0.48	0.04	0.94	0.71	0.014	0.37	0.85
T1	0.44	0.41	0.44	0.44	0.42	0.05	0.89	0.75		0.13	
T2	0.51	0.54	0.55	0.54	0.53	0.05	0.89	0.83		0.46	

^1^*n* = 1 animal/pen; 12 pens/tg; ^2^ NC = negative control; PC = positive control, NC + 20 mg amoxicillin/kg BW twice a day for the first 5 days of the feeding trial; 150 g/t; GE = NC + PFA grape extract, 150 g/t. ^3^ SEM Standard error of mean based on LSMeans. ^4^ T (sampling point): T1, day 27/28; T2, day 55/56, overall (T1 + T2). ^(a,b)^ Values within a row without a common superscript differ at *p* ≤ 0.10.

**Table 8:** Effect of using NC and PC for testing dietary GE supplementation on the concentrations of ammonia (µg/g) and biogenic amines (BA) (mmol/kg) in colonic content of weaning piglets at two different sampling points ^1^.

Item ^4^	Treatment Group (tg) ^2^			Sex		SEM ^3^	*p*-Value				
	NC	PC	GE	m	f		tg	Sex	T	tg × Sex	tg × T
Ammonia											
Overall	371	322	358	361	340	28.5	0.46	0.52	0.004	0.30	0.18
T1	464	353	383	385	416	44.6	0.21	0.56		0.48	
T2	278	291	333	337	264	35.4	0.52	0.085		0.41	
Total BA											
Overall	4.82	4.99	4.53	5.08	4.49	0.4	0.70	0.18	0.23	0.79	0.58
T1	4.84	5.57	4.76	5.02	4.24	0.6	0.59	0.72		0.84	
T2	4.81	4.42	4.33	4.98	4.06	0.4	0.73	0.083		0.92	
Ethanolamine											
Overall	0.58	0.55	0.51	0.57	0.53	0.04	0.45	0.33	0.001	0.023	0.61
T1	0.53	0.46	0.41	0.48	0.46	0.05	0.32	0.77		0.22	
T2	0.62	0.64	0.61	0.66	0.59	0.05	0.92	0.28		0.085	
Methylamin											
Overall	0.14	0.12	0.11	0.11	0.14	0.02	0.38	0.21	0.025	0.65	0.11
T1	0.19	0.16	0.10	0.16	0.14	0.03	0.15	0.50		0.92	
T2	0.10	0.09	0.11	0.11	0.09	0.02	0.65	0.19		0.52	
Pyrrolidin											
Overall	0.09	0.09	0.10	0.09	0.09	0.008	0.68	0.59	0.002	0.29	0.65
T1	0.08	0.08	0.08	0.07	0.08	0.005	0.99	0.28		0.024	
T2	0.11	0.09	0.11	0.11	0.10	0.01	0.62	0.30		0.27	
Putrescin											
Overall	0.63	0.64	0.60	0.67	0.58	0.06	0.80	0.22	0.14	0.82	0.59
T1	0.63	0.69	0.71	0.70	0.65	0.1	0.85	0.68		0.76	
T2	0.64	0.59	0.47	0.63	0.50	0.08	0.24	0.13		0.99	
Cadaverin											
Overall	1.70	1.79	1.44	1.79	1.50	0.3	0.65	0.37	0.042	0.98	0.61
T1	1.84	2.33	1.78	2.07	1.89	0.5	0.66	0.75		0.79	
T2	1.56	1.26	1.09	1.51	1.10	0.3	0.57	0.25		0.67	
Histamine											
Overall	0.37	0.44	0.39	0.41	0.38	0.04	0.44	0.55	0.009	0.95	0.15
T1	0.25	0.43	0.32	0.33	0.34	0.07	0.17	0.85		0.76	
T2	0.48	0.44	0.46	0.50	0.43	0.05	0.83	0.20		0.66	
Spermidine											
Overall	1.16	1.19	1.26	1.23	1.17	0.07	0.56	0.41	0.55	0.20	0.76
T1	1.15	1.20	1.20	1.16	1.20	0.09	0.89	0.74		0.94	
T2	1.17	1.19	1.32	1.31	1.12	0.09	0.48	0.14		0.025	
Spermine											
Overall	0.17	0.17	0.18	0.18	0.16	0.01	0.92	0.17	<0.001	0.38	0.55
T1	0.21	0.22	0.21	0.22	0.21	0.02	0.90	0.44		0.86	
T2	0.12	0.12	0.15	0.14	0.12	0.02	0.45	0.21		0.077	

^1^*n* = 1 animal/pen; 12 pens/tg; ^2^ NC = negative control; PC = positive control, NC + 20 mg amoxicillin/kg BW twice a day for the first 5 days of the feeding trial; 150 g/t; GE = NC + PFA grape extract, 150 g/t. ^3^ SEM Standard error of mean based on LSMeans. ^4^ T (sampling point): T1, day 27/28; T2, day 55/56, overall (T1 + T2).

## Data Availability

Not applicable.
